# Effect of donor and red blood cells concentrate characteristics on recipient hemoglobin increment following red blood cells transfusion in pediatric patients

**DOI:** 10.12669/pjms.38.6.5739

**Published:** 2022

**Authors:** Nazish Saqlain, Naghmana Mazher, Sundas Arshad, Maha Sajjal

**Affiliations:** 1 Dr. Nazish Saqlain, FCPS (Hematology). Department of Hematology and Transfusion Medicine, The University of Child Health Sciences & The Children’s Hospital, Lahore, Pakistan; 2 Dr. Naghmana Mazher, M.Phil. (Hematology) Pathology Department, Fatima Jinnah Medical University, Lahore, Pakistan; 3 Dr. Sundas Arshad, FCPS (Hematology). Department of Hematology and Transfusion Medicine, The University of Child Health Sciences & The Children’s Hospital, Lahore, Pakistan; 4 Miss Maha Sajjal (BSc. Medical Lab Technologist) Department of Hematology and Transfusion Medicine, The University of Child Health Sciences & The Children’s Hospital, Lahore, Pakistan

**Keywords:** Transfusion, Hemoglobin, Body Mass Index, Leukoreduction, Hematocrit

## Abstract

**Objectives:**

To determine the effects of donor and red blood cells concentrate characteristics on recipient hemoglobin increment following red blood cells transfusion in pediatric patients.

**Methods:**

This cross-sectional study was conducted at The Hematology & Transfusion Medicine Department of The UCHS & The Children’s Hospital, Lahore from 23^rd^ December 2020 to 31^st^ July 2021 after Institutional Ethical committee approval. After taking informed consent from parents/guardians, One hundred recipients receiving RBCs unit transfusion studied along with the respective donors. The donor’s details were recorded on a pre-designed proforma which included age, gender, Body Mass Index (BMI), CBC analysis (Hemoglobin [Hb] & Hematocrit) and blood group. Components’ preparation, storage and modifications details were also recorded. Hb levels of recipient were determined 12 hours prior to transfusion and 12-18 hours after transfusion. The data was analyzed on SPSS version 26.

**Results:**

Among recipients, the mean age was 5.25 ±3 years and male to female ratio was 1.16:1. The mean pre-transfusion Hb level of patients was 6.48g/dl (SD: 2.15) and mean post transfusion Hb was 8.824 g/dl (SD: 2.03) with a significant raise after transfusion (p< 0.001). Majority donors (60%) were between 18 to 30 years of age and mean age was 30.7 years (SD: 9.04). The hemoglobin increment was reduced for transfusion of RBC units from donor with greater age. Post- transfusion Hb rise was more in Rh D positive donations than Rh D negative (p< 0.0001). No significance of donors’ gender, BMI, Hb and hematocrit was found in relation to Hb increment. Among RBCs concentrate features, washing with normal saline found to have greater Hb increment, particularly in Thalassemia patients (p< 0.0001).

**Conclusion:**

Donors’ age and Rh blood group and red blood cell concentrates’ washing accounts for significant rise in recipient’s post-transfusion hemoglobin. These factors may be used to predict changes in recipients’ hemoglobin before transfusion.

## INTRODUCTION

Red blood cell (RBC) concentrate transfusion is a potentially lifesaving intervention, yet the harms, benefits and scarcity of blood have to be balanced.^1^ The transfusion of RBC concentrate is indicated in order to achieve a fast increase in supply of oxygen to the tissues, when the concentration of hemoglobin is low or oxygen carrying capacity is reduced and in the presence of inadequate physiological mechanisms of compensation.^2^

In pediatric patients, hemoglobin trigger demanding RBC transfusions varies according to the condition. Clinical assessment is vital. The usual dose of RBCs administered is 10-15 mL/kg leading to hemoglobin (Hb) increment of 2- 3 g/dL.^3^ The selection of healthy blood donors becomes more important in this regard to prevent alloimmunization and other adverse reactions. In Pakistan, a huge chunk of children suffers from inherited disorders like Thalassemia, hemophilia, rare bleeding disorders (deficiencies of coagulation factors as fibrinogen, Factor (F) FII, FV, FVII, combined FV/FVIII, FX, FXI, and FXIII) and Platelet function defects (Glanzmann Thrombasthenia, Bernard Soulier syndrome, Storage pool defects etc.) who require frequent transfusions as a part of their therapy.^4^,^5^

Blood is a unique medicine obtained by the courtesy of a healthy volunteer donor. So, donor selection and referral are an integral part in assurance of transfusion safety. Donor characteristics such as age, Body mass Index (BMI), Hemoglobin (Hb), Hematocrit, gender, blood group etc. can be the important determinants of a successful transfusion in terms of post transfusion hemoglobin increment.^6^ The final impact of packed RBCs preparation is determined by initial state of erythrocytes in the blood of a donor.

The RBC concentrates can be prepared from whole blood donation or by apheresis. They are stored at the temperature of 2-6 ºC for 35-42 days depending on the choice of anticoagulation agent used.^7^ The components may undergo a number of modifications as per need like leukoreduction, freezing, washing and irradiation. Gamma irradiation and prolonged storage of component have been linked with increased hemolysis in vitro.^8^ Among different centers, choice of additive solutions, method of collection, storage duration and transportation can vary. These factors can play their part in determining the outcome of RBCs unit transfusion. Some studies have calculated impact of pre- transfusion parameters and product quality on recipients’ outcomes, but such studies are less in pediatric age groups and have not been done in our region.^6^,^9^

In this study we have focused on pediatric patients only and tried to identify the donor and red blood cells concentrate features which can help select the best possible blood donors and blood bank procedures to attain maximum benefit after packed red cells transfusion. This benefit is measured in the form of post transfusion hemoglobin increment. This study would also link the gaps if any between pediatric and adult transfusion settings in this regard.

The objective was to determine the effects of donor and red blood cells concentrate characteristics on recipient hemoglobin increment following red blood cells transfusion in pediatric patients.

## METHODS

It was a cross-sectional study, carried out at The Hematology & Transfusion Medicine Department of The University of Child Health Sciences & The Children’s Hospital, Lahore from 23^rd^ December 2020 to 31^st^ July 2021 after Institutional Ethical committee approval [IRB# 1390/SAHS dated 22-12-2020]. After taking informed consent from parents/guardians, one hundred patients were randomly selected from indoor and outdoor units, who received RBC concentrate transfusion. The respective blood donors were also studied, identified, and traced back by the unique donation number. The donor’s details were recorded on a pre-designed proforma which included age, weight, height (for BMI calculation), Complete blood count analysis (Hb & Hematocrit) and blood group (ABO and Rh).

Component’s preparation method, date and time, storage conditions and modifications (Leukoreduction, gamma irradiation and washing) were also recorded. Leukoreduction was done by using bedside Baxter leucofilters and saline washing was done manually. Irradiation was done by using The Gammacell Irradiator. Manufacturers’ guidelines were followed for all procedures. Date and time of commencement of transfusion were noted at the respective clinical sites. Hb levels of recipients were determined 08-12 hours prior to transfusion and 12-18 hours after transfusion. Donors and recipients’ venous blood samples (3ml) were collected in vacutainers containing EDTA for CBC which were run on Sysmex XP/100. ABO (Forward and Reverse) and Rh blood grouping were done by tube method using EDTA and serum samples. Compatibility testing was performed through IAT (Indirect Anti-Human Globulin) tube method. Lorne Anti-sera A, B and D were used for blood grouping.

The data was analyzed on SPSS version 26. Qualitative variables were represented as frequencies and quantitative variables as mean and SD. Paired t- test was applied for comparing recipients’ pre-transfusion and post-transfusion Hb and hematocrit levels and independent t-test was used to compare mean Hb increment with categorical variables. P-value< 0.05 was taken as statistical significance.

## RESULTS

Among the one hundred recipients studied, the mean age was 5.25 ±3 years and male to female ratio was 1.16: 1. Majority donors (60%) were between 18 to 30 years of age and 40% were between 31 to 45 years of age, with mean age of 30.7 years (SD: 9.04). Male donors constituted 96%. BMI in the range of 17.1 to 25.7 kg/m^2^ was found in 56% donors with the mean value of 25kg/m^2^. B+ve (36%) was the commonest blood group and Rh positivity was found in 82% donors. (Table-I)

**Table I:** Clinical and demographic details of Donors.

Donors’ Characteristics	N= 100			
Age	18-30years: 60%	31-45years: 40%	Mean: 30.7 years (SD: 9.04)	
Gender	Male: 96%	Female: 4%		
BMI (kg/m^2^)	17.1 to 25.7: 56%	26.0 to 31: 44%	Mean:25kg/m^2^(SD: 3.26)	
Rh Status	Rh Positive	82%	Rh Negative	22%
ABO blood Groups				
A+ve	22%	A-ve	4%	
B+ve	36%	B-ve	8%	
AB+ve	8%	AB-ve	6%	
O+ve	12%	O-ve	4%	
Hemoglobin (g/dl)	12-13g/dl: 38%	14-15g/dl: 62%	Mean: 14.767	(SD: 1.2875)
Hematocrit (%)	Mean: 42.540	(SD: 4.8356)		

All RBCs units were prepared by whole blood centrifugation and 76% were transfused within seven days of storage. Among RBCs concentrate modifications, leucodepletion was done only for oncological (44) and ten Bone Marrow Transplant (BMT) patients while irradiated components were used only for BMT unit patients. Saline washing of RBCs units was done for 36 patients (34 Thalassemia major and two from Medical ICU) (Table-II).

**Table II:** Frequency of RBCs Concentrate modifications, preparation method and storage.

Components’ Characteristics	N= 100		
Method of Preparation	Whole blood Centrifugation: 100%	Apheresis: None	
Storage Duration	≤7 days= 76%	>7 days <35 days: 24%	Mean: 2.76days (SD:1.317)
Modifications	Washing: 36%	Leucodepletion: 54%	Irradiation: 10%

The mean pre-transfusion Hb and hematocrit level of patients were 6.48g/dl and 18.136% respectively while mean post-transfusion Hb and hematocrit level of patients were 8.824 g/dl and 26.324% respectively with a significant difference after transfusion (p< 0.001) (Table-III).

**Table III:** Patients’ Pre-Transfusion and Post-Transfusion Hb & Hematocrit Level.

Parameters	Mean	Std. Deviation	Correlation	p-Value
Pre-Transfusion Hb Level	6.483g/dl	2.1534	0.688	<0.001^*^
Post- Transfusion Hb Level	8.824g/dl	2.0317		
Pre-Transfusion Hematocrit Level	18.136%	5.6801	0.645	<0.001^*^
Post- Transfusion Hematocrit Level	26.324%	6.2947		

The hemoglobin increments were reduced for transfusion of RBC transfusion units from donor with greater age. No significance of donor’s BMI on Hb increment was found. The donor Hb level ranged from 12.5 to 17.5 g/dl with mean value of 14.7 g/dl (SD: 1.28). This Hb range resulted in required post transfusion increments. All transfusions were ABO and Rh fully compatible. ABO matched units showed the required Hb increment with no significant difference. Post- transfusion Hb rise was more in Rh D positive donations than Rh D negative units (p< 0.0001). Washing of RBCs units with normal saline found to have greater Hb increment (p< 0.0001). While blood irradiation and leucodepletion did not show significant variation (Table-IV).

**Table IV:** Correlation of Hb increment with Donors and Components’ characteristics.

Sr. #	Donors’ Characteristics	Hb Increment [g/dl] (Mean ± SD)	p-Value
1.	***Donor Age (Years)***		
	18-30 years	2.64±0.29	<0.0001^*^
	31-44 years	2.10±0.43	
2.	***Donor gender***		
	Male	2.44±0.44	0.5579
	Female	2.31±0.05	
3.	***Donor BMI (kg/m^2^)***		
	17.1 to 25.7	2.58±0.48	0.3030
	26.0 to 31	2.49±0.36	
4.	***Donor Hb Level (g/dl)***		
	12-13g/dl	2.39±0.42	0.2200
	14-15g/dl	2.50±0.44	
5.	***Donor Rh blood group***		
	Positive	2.57±0.31	< 0.0001^*^
	Negative	1.89±0.43	

*Sr. #*	*Components’ Characteristics*	*Hb Increment [g/dl] (Mean ± SD)*	*p-Value*

1.	***Method of Preparation***		
	Whole blood Centrifugation	2.42±0.45	
	Apheresis (None)	-	-
2.	***Storage Duration***		
	≤7 days	2.57±0.21	0.0547
	>7 days <35 days	2.45±0.39	
3.	***Modifications:***		
3.1	***Washing***		
	Yes	2.60±0.26	< 0.0001^*^
	No	2.32±0.49	
3.2	***Leucodepletion***		
	Yes	2.44±0.46	0.6498
	No	2.40±0.41	
3.3	***Irradiation***		
	Yes	2.35±0.28	0.3811
	No	2.47±0.42	

## DISCUSSION

Determination of impact of blood donors and red cells components’ features on post-transfusion outcome among pediatric patients in the form of measurable differences in hemoglobin increment can be helpful in selecting best possible units. These factors can be responsible for a considerable share of the variation seen clinically.

Starting from donors’ characteristics, donor age may affect transfusion outcomes in pediatric age group. In our study, 60% donors were between 18 to 30 years of age with mean age of 30.7 years (SD: 9.04). The hemoglobin increments were reduced for transfusion of RBC units from donor with greater age. Similar findings have been reported in other studies.^10^,^11^ Although these studies are from adult cohort so more extensive studies from pediatric group should be carried out from multiple centers in our country to guide transfusion policy in pediatric patients. An insufficient rise in erythropoietin to recompense aging marrow cells, loss of hematopoietic stem cells and iron deficiency due to dietary reasons particularly in our part of the world or because of frequent blood donations can be contributory factors in older blood donors.^6^,^12^

In our study majority were male donors with a meager contribution from female donors. The findings are similar to other study.^13^ No significant difference in Hb increment was found in relation to donor gender. In order to establish the cause-effect relationship a study with equal representation of both genders is of utmost importance which can lead to a convincing step to bring females to blood donation camps. Although, Roubinian NH et al have found that male derived blood donations have resulted in better Hb increment as compared to donations collected from females.^6^ This may be contributed to higher RBC mass in male donors. Chasse M. et al have favored male donors over females in terms of prevention of Transfusion related acute lung injury (TRALI).^14^

Post-transfusion Hb rise was more in Rh D positive donations than Rh D negative units, although only Rh compatible transfusions were considered. Our findings are similar to other study.^6^ This factor may be due to the presence of other favorable patient or disease related factors as reported in another study.^15^ The role of genetic polymorphisms in donors’ RBC genes need to be explored for better prediction of RBCs unit transfusion results.^6^ Donors’ BMI and ABO blood groups did not show any significant difference in transfusion outcomes. This may be due to the reasons that obese individuals were deferred as they didn’t fulfill the donors’ selection criteria. ABO fully compatible transfusions were studied and all types of major and bidirectional mismatch were excluded.

In the present study all components were prepared from whole blood centrifugation, so different methodologies of preparation cannot be compared. However, Barshtein G et al. studied RBCs deformities during packed cells preparation and concluded that exposure of RBCs to higher stress, during preparation, induces opposing effect so affecting post transfusion outcome.^16^

Among RBCs concentrate modifications, Leucodepletion was done only for oncology and Bone Marrow Transplant (BMT) patients while irradiated components were used only transplant patients within eight hours of irradiation. We did not find any significant relation of post transfusion Hb with these modifications. However, other studies have revealed that gamma irradiation was associated with less Hb increments in contrast to nonirradiated units.^17^ Irradiation can lead to disruption of RBC membrane resulting in increase in potassium.^18^ Saline washing of RBCs units was done for 36 patients. Washing with normal saline found to have greater Hb increment, particularly in Thalassemia patients (p< 0.001). Washing can also be helpful in reducing bacterial outgrowth and lung injury in critically sick patients.^19^

### Limitations of the study

It included incomplete documentation at clinical sites and required a meticulous follow-up of recipients after transfusion so keeping the number of participants low in a given time period. Such studies should be carried out for a longer duration and at different institutions to gather more comprehensive information which can guide the policy makers in terms of pediatric transfusions with maximum benefits. Moreover, in future research, study of recipient characteristics and cross linkage between donor, component and recipient factors is highly recommended.

## CONCLUSION

Donor characteristics such as age and Rh blood group and red blood cell concentrates’ washing accounts for significant rise in recipients’ post-transfusion hemoglobin. Collectively these factors account for significant variations in hemoglobin increment after transfusion among pediatric patients and may be used to predict changes in hemoglobin before transfusion.

### Authors’ Contribution:

**NS:** Conceived, designed, did statistical analysis & editing of manuscript, will be responsible, accountable for the accuracy and integrity of the work.

**MS & SA:** Did data collection and manuscript writing.

**NM:** Did review and final approval of manuscript.
